# Impact of Climate Change on Regulation of Genes Involved in Sex Determination and Fruit Production in Cucumber

**DOI:** 10.3390/plants12142651

**Published:** 2023-07-14

**Authors:** Aparna, Agnieszka Skarzyńska, Wojciech Pląder, Magdalena Pawełkowicz

**Affiliations:** Department of Plant Genetics, Breeding and Biotechnology, Institute of Biology, Warsaw University of Life Sciences, 02-776 Warsaw, Poland; aparna_aparna@sggw.edu.pl (A.); agnieszka_skarzynska@sggw.edu.pl (A.S.); wojciech_plader@sggw.edu.pl (W.P.)

**Keywords:** sex determination, flower development, fruit yield, climate change, ethylene, stress response, drought stress, high temperature stress

## Abstract

Environmental changes, both natural and anthropogenic, mainly related to rising temperatures and water scarcity, are clearly visible around the world. Climate change is important for crop production and is a major issue for the growth and productivity of cucumbers. Processes such as sex determination, flower morphogenesis and fruit development in cucumbers are highly sensitive to various forms of stress induced by climatic changes. It is noteworthy that many factors, including genetic factors, transcription factors, phytohormones and miRNAs, are crucial in regulating these processes and are themselves affected by climate change. Changes in the expression and activity of these factors have been observed as a consequence of climatic conditions. This review focuses primarily on exploring the effects of climate change and abiotic stresses, such as increasing temperature and drought, on the processes of sex determination, reproduction, and fruit development in cucumbers at the molecular level. In addition, it highlights the existing research gaps that need to be addressed in order to improve our understanding of the complex interactions between climate change and cucumber physiology. This, in turn, may lead to strategies to mitigate the adverse effects and enhance cucumber productivity in a changing climate.

## 1. Introduction

Agriculture in many ways is closely linked to changes in climatic conditions, whether natural or anthropogenic, as both have a significant impact on the yield of economically important vegetable crops, such as cucumbers. Due to the high concentration of nutrients, micronutrients, vitamins and minerals, cucumbers are essential sources for human nutrition. Favorable environmental growing conditions, which depend on many factors, are the most important criteria for good crop quality. These factors include good growing conditions, soil, cultivation techniques, irrigation and fertilization, as well as biotic and abiotic conditions, all of which have a major influence on quality of the cucumber. Due to continuous changes in climatic conditions, the quality of cucumbers could be affected [1,2]. Climate change is caused by increased emissions of greenhouse gases (carbon dioxide, nitric oxide and methane). Rising greenhouse gases not only increase global temperatures, but also alter rainfall patterns, humidity, drought and CO_2_ concentration. This could lead to reduced yields, which may not be enough to meet food needs as the world’s population continues to grow. Predicting and understanding the effects of climate change on plant developmental processes, from the molecular basis to the desired phenotype, is increasingly important.

**Figure 1:**
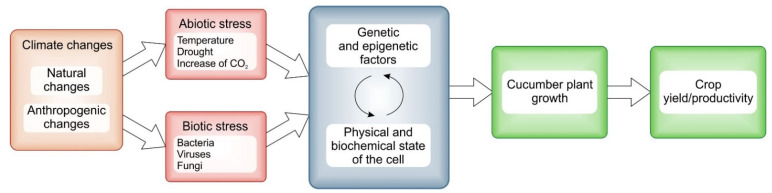
Schematic model of factors correlated with climate change and their impact on plant development and crop productivity.

Crops are exposed to abiotic and biotic stresses as a result of climate change. Abiotic stresses (temperature, drought and CO_2_ concentration) can have a direct effect on plant development. Increasing temperatures affect physiological and biochemical pathways that are essential for respiration, cellular mechanisms, photosynthetic activity and enzymatic activity, and they also influence the action of various genetic factors, transcription factors and hormones. This can affect changes in the genome and epigenetic regulation, which affect changes in the regulation of transcription and translation, causing physical and biochemical changes. Such a self-perpetuating condition can lead to the production of various plant defense mechanisms in response to changing climatic conditions. As a result, this affects the development of the plant and leads to changes in crop and yield productivity (Figure 1).

In addition, abiotic stresses can also affect biotic stresses. Biotic stresses resulting from pathogen attack can have direct or indirect effects on crop species. The direct effects of pests on plants include reproduction survival, development and spread of the pest, while the indirect effects include the relationship between the pest/pathogen to the environment and to other pest/organisms, such as competitors, enemyspecies, vectors and mutualists. Some common pest/pathogen of cucumbers include *Podosphaera xanthii*, *Tetranychus urticae* and *Aphis gossypii,* and some viruses include cucumber mosaic virus (CMV), cucumber green mottle mosaic virus (CGMMV), cucurbit yellow stunting disorder (CYSDV), potyviruses zucchini yellow mosaic virus (ZYMV), papaya ringspot virus (PRSV), watermelon mosaic virus (WMV) and Moroccan watermelon mosaic virus (MWMV) are very well studied for their interaction with cucumbers in changing climatic situation [3,4]. A coevolution of plants and pathogens can be observed in the environment. Changes in climatic conditions have accelerated this process and led to the evolution of new pests and pathogens, resulting in the introduction of new plant diseases [5]. Therefore, climate change poses a higher risk to vegetable crops, such as cucumbers.

The cucumber (*Cucumis sativus*, *n* = 7) belongs to the Cucurbitaceae family, which includes 825 species in 118 genera and is one of the most important economic crops [6,7]. Cucumbers are susceptible to various stresses, either biotic or abiotic, which severely affect agronomic factors, such as plant growth and productivity. The productivity of cucumber is largely dependent on the formation of good-quality fruits. Fruit development depends on the completion of two stages of the plant life cycle: the vegetative and the reproductive stages. Both of these stages are highly sensitive to environmental stress conditions. In cucumber cultivation, variable growth patterns are observed. This includes high fruit production with delayed growth as well as low fruit production with rapid growth [8]. Kahlen (2007) conducted a study that emphasized the effect of environmental conditions on plant growth [9]. The research highlighted the environmental factors, including temperature, light, humidity and nutrient availability, that significantly affected plant development and growth patterns. In addition, the architecture of a plant plays a crucial role in determining its growth and development which is largely dependent on the technical ability to cultivate.

Temperature is a major factor that changes continuously and is an important determinant of cucumber growth, development and yield. Higher temperature, both air and soil, reduces overall growth by affecting various physiological processes, such as reduced photosynthesis rate and increased transpiration rate [10,11]. Decreased photosynthesis results in reduced biomass and smaller fruit size, while increased transpiration rate results in increased plant water demand, leading to loss of soil water reserves and thus plant water stress. Combined temperature and water stress significantly reduce crop productivity, affecting flowering onset, male to female flower ratio, health and viability of male and female reproductive elements, reduced fruit yield and accelerated fruit ripening. The key factors, including temperature, light and relative humidity, also influence plant stomatal conductance, vapor pressure deficit and transpiration, which also have an impact on the production [12]. The temperature change also affects phytohormone (ethylene, auxin, GA and ABA) biosynthesis and signaling factors, thus altering processes at the molecular level, resulting in altered gene expression and further modification of the composition of the proteome and metabolome. This leads to developmental changes that affect yield quality. To cope with stress, plants have developed various stress response mechanisms, that activate molecular factors by regulating gene expression, involving specific transcription factors and signaling miRNA molecules.

The aim of this study is to identify possible pathways through which climate change may affect sex determination at the molecular level, which in turn affects fruit development and crop yield. Understanding how growth development and sex determination are regulated may lead to increased yields and improved agricultural practices. Our goal is to identify sensitive and critical points in the biology of cucumber flower development that may occur under changing climatic conditions and reduce yield productivity and quality. Finding solutions to increase crop productivity is essential to combat environmental impacts and meet the food needs of a rapidly growing population. Therefore, there is a need to focus on linking gene function to important agronomic traits as well as the development of genetic transformation techniques and gene-editing technologies for cucumbers, which are essential for functional studies on cucumbers.

## 2. Cucumber Growth, Flower and Fruit Development under the Impact of Climatic Changes

### 2.1. Basics of Cucumbers

The cucumber originates from tropical and subtropical regions; therefore, it prefers warm weather, and the optimal temperature for growth is 25–30 °C during the day and 18–21 °C at night. Nowadays, cucumbers are cultivated worldwide, but it requires a minimum of 15 °C for its development [13] and is sensitive to chilling and frost [14,15]. For good yield, cucumbers require high light intensity [16] and well-draining soils rich in organic matter at a pH of 6.0–7.0. The supply of cucumbers is limited due to several factors affecting production [17]. In cucumbers, the female flower development is particularly important, as it leads to fruit production and increases crop yield and productivity.

There are several sex morphotypes in cucumbers, including monoecious, dioecious, andromonoecious, trimonoecious, gynoecious and hermaphrodite [18]. These sex types are determined by the presence and arrangement of male and female flowers on the plant, making cucumber a good model for studying sex determination, fruit development and the vascular system in plants [19,20]. Flower development begins with the initiation of floral meristems and ends with blooming. In cucumbers, flower buds initially contain the potential to develop both male and female reproductive structures. However, the expression of certain genes and their interactions ultimately determine whether the flower will develop as male, female or hermaphroditic. Specifically, the inhibition of either the stamens or the carpels development results in the formation of unisexual flowers. In the absence of inhibition, the flower develops with both stamens and carpels, resulting in a bisexual flower [18,21].

The initiation of flowering and subsequent fruit development in cucumber plants is a complex process involving the interplay of multiple factors. Genetic elements, including transcription factors, repressors, genes and phytohormonal signals, play a crucial role in these processes. However, it is important to note that the expression and regulation of these genetic factors are controlled by the dynamic environmental conditions in which the plants grow. The combined action of these factors results in changes at multiple levels, including the physiological, morphological and molecular levels, which ultimately control the intricate processes involved in flower and fruit formation in cucumber plants. Both external and internal factors are important in determining how cucumber plants grow and develop.

### 2.2. Climatic Impact

Several interrelated phenomena affecting plant growth and yield are associated with the observed climatic changes. Increasing temperatures are correlated with a decrease in rainfall, which is associated with a decrease in the amount of water available, leading to drought stress. In addition, it affects the change of seasons and can lead to rapid changes in weather that negatively affect crops (frost, heavy or prolonged rainfall leading to flooding, long periods of drought). Therefore, in the present study, we paid more attention to two climatic factors, increasing temperature and drought, and their effects on the cucumber plants development, mainly in relation to sex determination.

#### 2.2.1. Effect of Temperature Increase on Cucumber

Climate change can be described as a long-term, significant difference in average weather [22]. Like other agricultural crops, cucumber production also depends on environmental/climatic conditions for a better yield. In general, farmers face losses in the form of low productivity due to climate change [22,23,24]. This temperature variation can affect vegetable crops. As the optimum temperature for cucumber growth is between 20 and 25 °C, temperatures of around 35 °C can induce heat stress in the plants [25]. Stress has been well studied for reducing crop production and accelerating fruit ripening, which affects fruit quality [26]. Elevated temperature suppresses photosynthetic processes by modulating enzymatic activity, mainly Rubisco and other related enzymes [10] and the electron transport chain [27], resulting in the impairment of chlorophyll biosynthesis [11]. High temperature can also indirectly affect the photosynthetic process by increasing leaf surface temperature and affecting stomatal conductance [28,29]. Elevated temperatures not only affect the above ground parts of plants, but also significantly affect the root system. Heat in the root zone significantly reduces plant height, stem diameter, shoot fresh weight, shoot dry mass, and shoot water content of cucumbers.

The duration of initiation and expansion of floral organs and leaves is shortened by warming, leaving less time for biomass accumulation, ultimately resulting in reduced plant size [30,31]. Higher temperatures (42 °C) also suppress seed germination in cucumbers, thereby reducing plant growth and reproductive traits. In cucumbers, increasing temperature has a detrimental effect on sex expression, flowering, pollination and fruit set. High temperatures and long days tend to keep the vines in the male phase, while short days and low temperatures encourage more female flowers. Thus, an increase in temperature may reduce the number of female flowers, which indicates lower productivity [32,33]; male flowers are increased, but they are smaller and have reduced nectar, which will affect pollination, and pollen per flower also decline. In addition, early flowers in cucumber may drop when exposed to extremely high temperatures [34,35]. At the stage of fruit development, if the cucumber plant is exposed to high temperature, it will cause bitterness in the fruit [34]. High temperatures exert significant effects on various physiological characteristics in cucumbers, including changes in malondialdehyde (MDA) content and the activities of catalase (CAT), peroxidase (POD), and superoxide dismutase (SOD) enzymes [11,36]. These studies provide scientific evidence for the effects of high temperature on cucumber physiology [37,38]. In addition, high temperatures have been found to disrupt normal flower development in cucumbers by inducing pollen sterility [36]. Leaf wilting, physical damage to plant shoot and root growth, physiological disorders, biochemical changes and reproductive problems also result in a significant reduction in crop yield at high temperatures [39,40].

#### 2.2.2. Impact of Drought on Cucumber

Drought is another major limiting factor for agricultural crops and, when combined with high temperatures, can affect the vitality of crops [41]. Depending on the species, yield losses under drought stress can range from 30 to 90 percent for sensitive crops. [42]. If plants can adapt their physiology to a drought environment, they will have a better chance of surviving. Higher temperatures may increase the ability of the environment to absorb more water vapor, resulting in more evapotranspiration by plants, which will lead to higher water requirements. Increase plant water demand could deplete the reservoir of water in the cultivating soil, creating a state of plant water stress (EPA, 2021). In dry and semidry climates, drought stress is the major factor that negatively affects plant growth and production, and undoubtedly reduces crop productivity [43,44,45,46,47]. Drought stress can reduce agricultural production by decreasing the activity of enzymes involved in the Calvin cycle [48,49,50].

The presence of drought results in numerous physiological, biochemical, morphological and molecular changes [43,44], including vascular tissue contraction, decreased water uptake [51] and impaired photo assimilate translocation [52]. In addition, drought inhibits ion uptake, impairs ATP biosynthesis and ROS accumulation, which accelerates oxidative damage and ultimately reduces plant development [53]. According to González Villagra et al. [54], drought stress disrupts the production of endogenous phytohormones by increasing ABA concentrations, decreasing IAA and GAs, and rapidly decreasing zeatin concentrations. The hormonal imbalance slows down the growth of plant cells by reducing their turgor, elongation and volume, leading to a decrease in growth characteristics [51]. Liu et al. (2018) conducted a study that demonstrated the negative effects of drought on cucumber seedlings, specifically showing a decrease in leaf thickness [55]. This can be attributed to a simultaneous decrease in the thickness of both the palisade and spongy layers. The study provides evidence of drought effects on cucumber seedling morphology. In addition, an Indian research group conducted a descriptive study on different cucumber genotypes and showed that drought also leads to a reduction in fruit yield per vine [56]. The study highlighted that different cucumber genotypes have different responses to drought stress. Farag et al. (2019) conducted a study that focused on the effect of drought stress on cucumber yield and its components [56]. The results of the study showed a significant decrease in yield, including a reduction in the number of fruits per plant, fruit weight per plant, and total yield, when compared to well-watered plants.

In addition, severe drought stress was found to have extensive detrimental effects on various aspects of cucumber growth and development. Metwaly et al. (2022) reported that severe drought significantly reduced vine length, fresh leaf weight, number of branches per plant, leaf number per plant, photosynthetic pigment content, and leaf area per plant [57]. These results provide evidence for the widespread negative effects of severe drought on cucumber plants. The very first response of plants to drought is stomatal closure. Further, as drought stress continues, plants induce other acclimation responses, such as cell wall modification and antioxidant production [58,59].

## 3. Molecular Regulation of Sex Determination, Flower and Fruit Development

Cucumbers are a species within the angiosperm family with the advantage of having perfect flowers with separate carpels and stamen coexisting together. The regulation of flower development, particularly of the female flower, has a significant impact on cucumber yield.

### 3.1. Sex Determination and Flower Morphogenesis Processes

Flowering is a critical trait in plants as it plays an important role in increasing crop productivity. Identifying and studying the genes involved in this process enables targeted approaches to crop improvement and agricultural sustainability.

**Figure 2:**
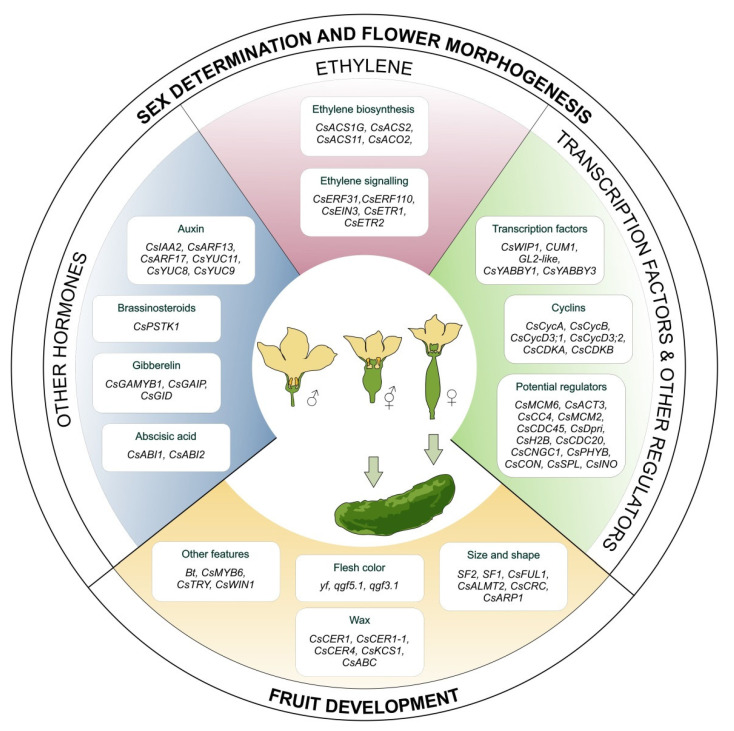
Genes and transcription factors related to the sex determination, flower morphogenesis and fruit development processes in cucumbers.

Several studies have reported the effects of phytohormones, genetic factors, and environmental factors on sex determination in cucumbers (Figure 2). At the phytohormonal level, ethylene serves as the primary hormone that facilitates sex determination in cucumber flowers [60,61,62]. Auxin, cytokinin and brassinosteroids have a feminizing effect by interacting with ethylene signaling [63,64]. Although several ethylene synthesis genes involved in sex determination in cucurbits have been described, the focus is on elucidating the molecular link between the transition from male to female flowers or vice versa, which is still unknown.

**Figure 3:**
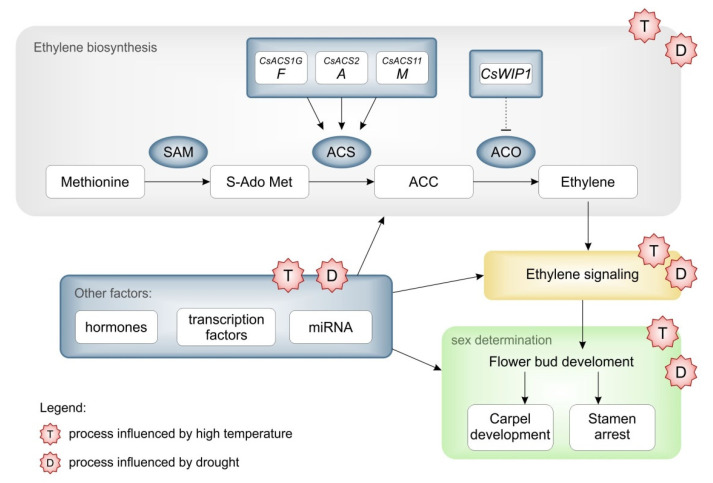
The hypothetical scheme of how the temperature and drought influence the sex determination in cucumbers. The scheme of ethylene biosynthesis and signaling in sex determination pathway in cucumbers, major molecular regulators of these processes and possible influence of climatic factors.

Ethylene synthesis is a two-step process that begins with the conversion of S-adenosyl-L-methionine (SAM) to 1-aminocyclopropane-1-carboxylic acid (ACC) by the enzyme ACC synthase (ACS) (Figure 3). ACC is then converted to ethylene by ACC oxidase (ACO) enzymes [65,66,67]. This conversion is regulated by three main genes: female (*F*, encoding an extra copy of the *CsACS1G* gene), androecious (*a*, encoding ACS11, which blocks the female flower developmental pathway), and monoecious (*M*, encoding *ACS2* in the carpel primordia) [68,69,70,71,72]. These genes encode ACC synthases and oxidases, that together regulate a complex ethylene synthesis process, leading to female flower development [73,74].

*CsACS1G*, *CsACS2*, *CsACS11* and *CsACO2* are some of the important genes involved in the synthesis of the ethylene in cucumber [75]. The *F* locus is responsible for femaleness and determines the female phenotype among the three genetic loci. In cucumber, the *F* gene refers to *CsACS1*, indicating a gynoecious plant [76]. A duplication of the *F* gene leads to the development of the *ACS1G* gene, which has a new promoter and is expressed early in the development of floral buds [77,78,79]. Even in the absence of *ACS11*, *ACS1G* regulates ethylene development in conjunction with *ACO2* [77,78]. Additionally, *CsWIP1*, which encodes a C2H2 zinc finger transcription factor, inhibits female flower development and leads to the formation of male flowers in cucumbers [80,81]. *CsWIP1* binds to the promoter of *CsACO2* and causes a decrease in its expression [74].

Yamasaki et al. (2017) observed a correlation between sex determination and cell cycle pathways in cucumbers [82]. They examined the expression of six cell-cycle-related genes: four *cyclins, CsCycA, CsCycB, CsCycD3;1* and *CsCycD3;2*; and two *cyclin-dependent kinases, A* (*CsCDKA)* and *CsCDKB* in male and female flower buds. Wang et al. (2019) performed RNA sequencing on young apical buds of gynoecious and female cucumber at three different growth stages [83]. They identified nine genes as potential candidates for sex differentiation regulators in cucumber: *Cs-MCM6*, *Cs-ACT3*, *Cs-XRCC4*, *Cs-MCM2*, *Cs-CDC45*, *Cs-Dpri*, *Cs-H2B*, *Cs-CDC20* and *Cs-CNGC1*. Five of them (*Cs-MCM6*, *Cs-MCM2*, *Cs-CDC45*, *Cs-Dpri* and *Cs-CDC20)* were found to be involved in the cell cycle pathway, suggesting that they may play an important role in cucumber sex determination [83]. A recent discovery has revealed the presence of an insertional LTR-RT mutation within the first exon of the *CsPHYB* gene, which is responsible for the long hypocotyl and early flowering phenotype observed in cucumbers [84].

Although ethylene-synthesizing genes are known to be involved in sex determination, some ethylene-signaling genes are also essential for this process. Following ethylene biosynthesis, little information is known about downstream ethylene-signaling to regulate female flower development. After the synthesis of ethylene, its signaling is regulated by several receptor proteins presented on the membrane of the endoplasmic reticulum (ER). In the presence of ethylene, several ethylene-responsive factors (*ERFs*) are activated and initiate the expression of several downstream ethylene-responsive genes [85,86,87]. An increase in the promoter activity of *CsACS11* through interactions with *CsERF110* in cucumbers was confirmed using yeast one-hybrid assay. This interaction also regulates ethylene signaling [88]. *CsEIN3* activates *CsERF31*, which stimulates *CsACS2* and acts as a positive feedback loop to increase ethylene and female-flower production in the ethylene signaling. *CsERF31* also responds to the *F*-derived ethylene signal and positively regulates ethylene feedback in cucumbers by activating *M* expression [89]. Ethylene perception interferes with stamen growth in female cucumber flowers through DNA damage. The ethylene receptor *CsETR1* is downregulated in the stamen of female flowers, causing stamen development arrest [90,91]. Under increased amount of endogenous ethylene, *CsETR2* and *CsERS* play role in female flower development in gynoecious cucumber plants. Molecular and functional characterization of two *C. pepo* mutants with altered ethylene receptors, *CpETR1A* and *CpETR2B*, indicated that mutations in these genes leads to the change of sex morphotypes, converting monoecious plants to andromonoecious plants. In addition to the initial hermaphroditization of female flowers, the number of male flowers also increase significantly [92,93]. Another ethylene-inducible DNase gene, *CsCaN*, functions in the stamen primordia of female floral buds [94]. This could be the reason for the inhibition of stamen formation in female flowers [91].

### 3.2. Factors Regulating Sex Determination

Ethylene is the main sex-related hormone, but other phytohormones, such as cytokinin, brassinosteroids and auxin, promote carpel development through interactions with ethylene, while gibberellins promote stamen development. In addition to ethylene, auxin indirectly influences the sex of cucumbers. Exogenous IAA can increase *ACS* gene expression and promote *ACC* and ethylene production, thereby influencing female flower formation [63]. *CsIAA2* and other auxin-inducible genes have been proposed to play a role in the promoting ethylene and thus regulating sex in cucumbers [95]. More female flowers were produced in both female and male lines directly due to an increase in exogenous auxin concentration. The auxin-induced binding of *ESR2* to the *ACS2* promoter increases the expression of *ACS2*. This binding increases more ethylene production, which in turn makes the cucumber more feminine [96]. In some cases, reduction in auxin biosynthesis had no effect on sex determination in cucumber, which may indicate that auxin synthesis does not play a direct role in sex expression [62]. Analysis of the cucumber MADS box (*CUM1*) suggests that auxin and ethylene regulate the development of male and female flowers by altering *CUM1* expression. This regulation of sex determination by auxins is mediated by the auxin response factors *CsARF13* and *CsARF17*, which act as upstream regulators of *CUM1* [97]. Auxin biosynthesis is regulated by *YUCs,* which catalyze the rate limiting step, and *CsYUC11,* which have specific expression in the stamen of male flower development [98]. The endogenous concentration of indole-3-acetic acid (IAA) serves as a defense mechanism against heat-induced damage by triggering the activation of *CsYUC8* and *CsYUC9* genes in response to high temperatures [98].

Cucumber plants treated with exogenous GA inhibit ethylene biosynthesis by suppressing the expression of the *CsACS1G* gene [62], which inhibits female flower production and promotes maleness. *CsGAMYB1*, a positive regulator of gibberellin signaling, is thought to produce male flowers while inhibiting female flowers [99]. *CsGAIP*, a negative regulator of GA signaling, inhibits B-class floral homeotic genes, thereby preventing the formation of male flowers. The ubiquitin proteosomal proteolysis of *CsGAIP* reduces B-class homeotic gene inhibition and promotes staminate development [100]. In addition, transcriptome analysis revealed that GA regulates cucumber sex expression in an ethylene-dependent manner, probably involving the genes *CsACS2*, *CsETR1* and *ERFs*, or in an ethylene-independent manner, involving the C-class floral homeotic gene *CAG2* [101]. High temperatures induce GA and GA-biosynthesis-related genes in *Arabidopsis* and soybeans. It is possible that high temperature induces male flower development by increasing GA, but this needs to be investigated [102]. Fukuda (2009) reported that elevated temperature also promotes bolting in lettuce by increasing the expression of *LsGA3ox1*, which is a key gene responsible for stem elongation [103].

Brassinosteroids also have an ethylene-dependent effect. Female flower production was increased by the application of epibrassinolide in cucumbers, melons and zucchinis [63,64]. Exogenous brassinosteroids increase cucumber femaleness and cause increased ethylene production [104]. BAK1, a receptor in the BR signaling pathway, and *CsPSTK1*, a putative serine/threonine kinase, are correlated, suggesting that *CsPSTK1* may be involved in BR signaling [105].

The role of ABA in sex determination has not been widely reported, but one study has identified *CsABI1* and *CsABI2* genes (members of the protein phosphatase 2C gene family) that are differentially expressed in male and female floral buds [106]. This finding suggests that ABA signaling is involved in the formation of male and female flowers and has a regulatory role during floral morphogenesis and a role in the selective development of specific whorls in unisexual flowers [106]. In addition to these parameters, hormones have complex interactions with sugars to maintain signaling pathways, particularly ABA and ethylene [107,108].

Plant growth and development is highly dependent on the coordinated expression of all above mentioned genes and many others that are regulated by plant transcription factors (TFs). These TFs can influence the expression of downstream genes by directly or indirectly binding to cis-regulatory elements in the promoter region of the target genes. They can upregulate or downregulate gene expression, and possess various motifs, including DNA-binding and activation motifs. There are several families of transcription factors with different motifs in their protein structure, such as bZIP, HD-ZIP III, NAC, AP2/EREBP, WRKY, bHLH, TCP, MADS Box, ARF and MYB families [109]. For example, the HD-ZIP IV family transcription factor *GL2-LIKE* interacts with *CsJAZ1* to form a complex that regulates male flower development, seed viability and pollen intensity by regulating the expression of the FT gene expression [110]. In addition, in cucumbers, *CsSPOROCYTELESS* (*CsSPL*) directly interacts with *CsYABBY1*, *CsYABBY3* and *CsINO* to regulate integument development in cucumber ovules, as evidenced by yeast two-hybrid (Y2H) assay [111].

The research reported above provides strong evidence for the association of phytohormones and transcription factors with variation in climate conditions for the regulation of flower morphogenesis in plants. Considering this, it can be said that factors such as temperature and drought are important in regulating flowering in plants.

### 3.3. Climate Effect on Flowering

The above mentioned genes may be related to climate change. Climatic conditions (drought, heat stress and humidity) have a major impact on the molecular mechanisms of the cell. This, in turn, changes the phenotypes of the flower [112,113,114].

Not many genes are identified in cucumber that could be affected by stress but are reported in other plant species. Temperature may play a role in activating or repressing *ACS* activity, resulting in either induced or suppressed ethylene production. It is accelerated in kiwis [115] and gets suppressed in tomatoes [116]. For cucumbers, whether it is suppressed or enhanced is still an unexplored, but it would be interesting to find out whether ethylene is suppressed because a reduction of female flowering is reported for cucumbers at high temperatures.

Not only high temperature, but also drought stress, is also related to ethylene signaling. Under drought conditions, ethylene signaling is severely affected in many plants [117,118,119]. Drought-induced ethylene biosynthesis leads to the de novo synthesis of *ACS*, which accumulates *ACC* and increases the ethylene levels [120]. In rice, *OsERF101* is reported to be induced by drought stress especially in reproductive structures [121]. Other genes, such as *OsERF109* and *OsERF3,* act as a potential genetic factor that improves drought tolerance by repressing ethylene signaling and the expression of ethylene biosynthetic genes, respectively [122,123]. A study on sugarcane demonstrated the role of *ERF3* in increasing tolerance to drought stress [124]. *OsARD1*, a metalloenzyme known as *ACIREDUCTONE DIOXYGENASE*, interacts with Fe^2+^ and mediates the synthesis of methionine, a critical precursor in the ethylene biosynthetic pathway. The overexpression of *OsARD1* in rice plants significantly increased endogenous ethylene levels, improved water capacity and reduced susceptibility to drought stress [125]. The effects of drought on sex expression and flower have not been well studied in cucumbers, so it would be very interesting to investigate the effects of drought on the sex expression and flower development in cucumbers. In addition, investigating the relationship between climatic conditions and the regulatory mechanisms of sex-related-genes are of a great interest in the context of cucumber research.

High temperatures are usually an inducer of high ethylene production due to the acceleration of all plant metabolic processes. High temperatures induce ethylene production in several agricultural crops, such as kiwi (*Actinidia deliciosa* (A. Chev.) [115,126], soybean (*Glycine max*) [127] and wheat (*Triticum aestivum* L.) [128]. In addition, molecular studies on soybeans have shown that an increase in ethylene production induced by high temperatures is associated with a decrease in photosynthetic activity and an increase in lipid peroxidation and reactive oxygen species accumulation [129].

Drought-tolerant plants respond to stress by increasing ethylene production. Alfalfa (*Medicago sativa* L.) [130], cotton [131] and rice [120] have been shown to increase ethylene production during a water deficit. Furthermore, a water deficit in wheat resulted in the accumulation of 1-aminocyclopropane-1-carboxylic acid (ACC) [120].

Since it has been shown in plants that drought stress can induce increased ethylene production, it is very likely that in cucumbers, depending on the genotype, this stress can affect the distribution of flowers on the plant or even the proportion in their appearance, with a tendency towards female flowers. In other plants, considering the regulation at the molecular level, studies of the transcriptome carried out under drought conditions allowed for the observation of an increased activity of genes related to ethylene biosynthesis and signaling in soybeans [132].

Ethylene can be considered as a marker of stress conditions. However, it can also have negative effects on production quality, for example, in ornamental plants. Ethylene induced by water stress during cultivation has been shown to increase the malformation of buds or flowers and negatively affect the quality of freesia [133,134].

### 3.4. Fruit Development and Regulators of this Process

The initiation of female flower formation marks a crucial moment in fruit development, and it is essential to note that the growing conditions during this phase are of a great importance. The interplay between environmental factors and the intricate biological mechanisms involved in fruit growth can significantly affect the final outcome. In addition to flower development, fruit growth depends on the interaction of genetic factors that can act as a growth inhibitors or stimulators. The interaction of various factors results in fruit diversity in terms of most basic morphological characteristics, such as shape, size and color, which are important criteria to improve fruit quality.

The size and shape of the fruit are remarkable features of cell division, which increases the number and proliferation of the cells. Cucumber fruits can have different shapes like long or short and oblong/oval/cylindrical, but the majority of cucumbers has an elongated shape. Various genetic factors involved in cell cycle pathway have a direct impact on the size of cucumber fruit. Recent research has shed light on the vital role of the *SF2* gene, which encodes a histone deacetylase HDC1 that plays a crucial role in regulating the proliferation of fruit cells by controlling the synthesis and metabolism of polyamines and cytokinins. *SF2* mutations result in a significant decrease in cell proliferation rate, which causes shorter cucumber fruit production [135]. Another gene that has been shown to affect cucumber fruit development is *SF1*, a RING-type E3 ligase. *SF1* mutations result in increased self-ubiquitination and degradation. This also leads to the accumulation of *ACS2,* which is a rate-limiting enzyme in ethylene biosynthesis. This overproduction of ethylene, in turn, leads to the development of shorter cucumbers in these mutants, showing a dose-dependent effect of ethylene [136]. *CsFUL1^a^*, a MADS-box like gene that binds to the promoter of Superman (*SUP*) and represses its expression, which can affect cell division and inhibit auxin transport during fruit elongation, causes the development of shorter fruits [137]. A newly discovered gene, *CsALMT2*, has been identified as being responsible for the formation of the hollow trait in cucumber. This gene is mainly expressed in the ovule development zone, which is located inside the fruit. The expression of *CsALMT2* in this specific zone suggests its involvement in regulating cellular processes related to ovule development, potentially affecting the overall fruit morphology, but the molecular mechanisms are still unidentified [138].

Fruit development in plants is influenced by phytohormones. GA and auxin positively regulate fruit development, while it is negatively regulated by ethylene [139,140,141]. The commercial value of cucumber fruit depends on its size, color and curvature. The overproduction of ethylene results in long fruits, while decreased ethylene production suppresses cell division, resulting in shorter fruits. Therefore, maintaining the correct ethylene dosage is critical to properly regulate cell division in the developing fruit [136]. The GA receptor gene, *CsGID1a*, is associated with fruit locule formation and therefore promotes cell expansion to control fruit shape in cucumber [142]. The amount of IAA affects fruit size at different developmental stages in cucumber. *CsYUC10b*, an auxin biosynthesis gene, is involved in the formation of fruit curvature, and its overexpression promotes straight fruit development [143].

In addition to their role in sex determination, the effects of bZIP family transcription factors have been reported in many processes, such as organ and tissue differentiation, hormone signaling, light response and pathogen defense in plants. They also include developmental processes, physiological systems and stress situations in different plants, such as maize [144], cucumbers [145] and tomatoes [146]. Two MYB genes, *CsMYB6* and *CsTRY*, negatively regulate trichome initiation on cucumber fruits [147], while *CsMYB60* regulates fruit spine color in cucumber [148]. In *Arabidopsis*, *WIN1* was identified as a wax-regulator gene and was found to be responsible for the glossy phenotype. *CsWIN1*, another AP2/ERF-type transcription factor, is associated with pericarp formation in cucumbers and increases the expression of wax biosynthetic genes, such as *CsCER1*, *CsCER1-1*, *CsCER4*, *CsKCS1* and *CsABC*, the wax transporter gene [149]. It has been observed that a member of the YABBY family called *CRC* (CRABS CLAW) influences fruit length in cucumbers. This gene has two alleles, namely *CsCRC^A^* and *CsCRC^G^*, which contribute to different phenotypes associated with short and long fruit length, respectively [150].

The downstream effects of *CsCRC* on fruit length involve the regulation of several genes involved in cucumber development. *CsARP1*, which encodes an auxin-responsive protein, serves as a target gene of *CsCRC* and plays an important role in promoting fruit elongation, primarily by facilitating cell expansion [150].

Fruits in cucumber are comprised of three distinct layers: epicarp, mesocarp and endocarp. The mesocarp and endocarp are responsible for the flesh of the fruit, which has a variety of colors ranging from orange to yellow and green or white. The main pigments that give the fruit its color are chlorophylls and carotenoids. β-carotene, as a vital nutrient, contributes significantly to maintaining healthy eyes. According to Cuevas (2010), the amount of β-carotene in the mesocarp and endocarp was controlled by two recessive genes and a single recessive gene, respectively. In cucumbers, the accumulation of β-carotene to high levels in the endocarp causes a natural genetic variant in a β-carotene hydroxylase gene [151]. Lu et al. (2015) identified a single recessive gene, *yf*, which is responsible for the yellow color of flesh in cucumber [152]. In addition, two loci, qgf5.1 and qgf3.1, responsible for the formation of green flesh in cucumber were identified through QTL mapping and GWAS. The candidate gene for qgf5.1 was reported to be *Csa5G021320* [153]. However, very little information has been reported on the molecular mechanism underlying the genetic factors that govern the regulation of fruit flesh color in cucumbers.

The effect of bitterness in fruits not only reduces their flavor but also diminishes their marketability, which ultimately leads to a decrease in demand for the crop and loss of profit for the farmers. The major component responsible for the bitterness in cucumber fruit is cucurbitacin C (CuC) [154,155], which is an oxygenated tetracyclic triterpenoid. The *Bt* (*bitter fruit*) gene, a transcription factor that regulates CuC biosynthesis, has been shown to control the levels of cucurbitacin only in cucumber fruits [156]. CuC is rapidly synthesized in response to biotic or environmental stresses, such as very low or very high temperature, drought or insufficient light intensity. Therefore, changes in the environmental conditions can affect fruit flavor and further the quality of market product [157]. Climatic conditions have a significant impact on the proper development of flowers and fruits. Thus, understanding the influence of climatic conditions on this critical stage can have impressive results on fruit quality and yield. However, little is known about the effects of climate change on fruit formation and development at the molecular level.

### 3.5. Regulation of mRNA and miRNA during Growth and Development by Climatic Factors

Transcriptome analysis revealed several key genes that are involved in the environmental stress response in cucumbers [158,159]. A high expression of NAC, abscisic acid 80-hydroxylase1, ethylene responsive genes, three WRKYs TFs, *CsCaM3* and bHLH96 are reported to have higher expression in heat stress condition to provide tolerance to high temperature in cucumbers [160]. It has been reported that most of the genes activated during heat stress encode transcription factors or coactivators involved in thermo-tolerance regulation. The key regulators of plant response to elevated temperature are heat shock factors (HSFs) [161]. In addition to these reported TFs, lncRNAs, circRNAs and miRNAs also play an important role in the heat stress response in cucumbers [162]. Antioxidant enzymes, such as SOD, CAT, APX and POD, also play a role in the heat stress response as well. They are more activated under stress and are involved in thermo-tolerance in cucurbits [163,164], and genes encoding these enzymes show higher expression under heat stress conditions also in cucumbers [165]. The HSP gene family, which encodes heat shock proteins, is of great importance in the response to heat stress, which has been well described in cucurbits. The role of the *HSP20* genes in response to elevated temperature has been well studied in pumpkins, cucumbers, melons and watermelons [166,167,168,169]. Thermo-tolerance is an important quantitative trait in cucumbers and is controlled by *QTLs.* Several articles have reported the role of QTLs in cucumbers under heat stress. QTLs-*qHT3.2* and *qHT1.1* in cucumber seedlings have been reported to tolerate heat stress [170,171].

As key regulatory molecules, miRNAs play a pivotal role in modulating gene expression or repression by directly binding to their target genes. The expression of miRNAs is also affected by abiotic and biotic stresses, such as drought, heat and CO_2_ [172]. In various plant species, miRNAs have been reported to be directly or indirectly associated with key factors involved in flower and fruit development processes, including phytohormones, in particular ethylene in cucumbers. In *Poncirus trifoliate*, the positive regulation of cold tolerance by miR396b occurs through the downregulation of *ACO* transcripts, resulting in the inhibition of ethylene synthesis and the facilitation of polyamine synthesis. This dual effect ultimately enhances the ability to scavenge reactive oxygen species (ROS) [173].

A comprehensive understanding of miRNA contribution to flower development was obtained by profiling miRNA expression in tomatoes. Potential miRNAs and their target genes that can be correlated with heat stress regulation were identified. miRNA–target pairs, such as miR398b-3p/*SlCSD1*, miR393-5p/*SlTIR1*, miR160a/S*lARF10/16*, miR156e-5p/*SlSPL15* and miR397-5p/*LACs,* were shown to be correlated with the regulation of heat stress response and metabolic pathways in stamens and pistils in tomatoes [174]. The other study showed the differential expression of miR156/157 in cucumbers under higher temperature conditions and negative correlation with the expression of its target genes, encoding SBP-box transcription factors [175]. It has been reported that SBP-box genes are crucial in the regulation of plant growth, flowering, fruit development and other processes [176]. Therefore, it can be hypothesized that miR156/157 regulates the physiological processes involved in plant growth and development through the SBP-box TFs. In addition to temperature, the effect of CO_2_ stress on flowering was shown to be mediated via miRNA in leaves and bolters of *Arabidopsis*. The expression of miR156/157 and miR172 is differentially regulated by high CO_2_ levels and elevated temperatures, showing different responses. High CO_2_ level facilitates an enhanced flowering process by suppressing the expression of miR156/157, which targets *SPL9*, while concurrently activating the expression of miR172, which controls the expression of AP2. Conversely, high temperature exerts an opposite effect on these miRNAs compared to high CO_2_ levels [177,178]. Furthermore, it has been observed that the expression of miR156 and miR172 is affected by temperature, leading to temperature-dependent effects on the *DOG1* gene. The *DOG1* gene plays a crucial role in regulating seed germination and flowering time, which are important developmental milestones in plants. In the case of lettuce, the downregulation of *LsDOG1* expression at high temperatures has been shown to facilitate seed germination and also promote early flowering. These effects are accompanied by a decrease in miR156 levels and an increase in miR172 levels [179].

Extensive research has revealed the important role of miR159 in *Arabidopsis*, rice and wheat, particularly in the regulation of the GAMYB-like family of transcription factors. These transcription factors play a critical role not only in flower development but also in the intricate gibberellin (GA) signaling pathways. In addition, GAMYB transcription factors were found to be involved in anther development. This shows that TamiR159 in wheat is involved in heat stress signaling, which in turn also affects anther development [180,181]. An analysis in cucumbers showed that csa-miR159b plays a critical role in ABA-mediated heat tolerance. During heat stress, ABA accumulates in plants and represses the csa-miR159b expression, which in turn causes the induction of the *CsGAMYB1* gene, which promotes male flowers development in cucumber (in an ethylene-dependent or -independent manner) [182].

In the study by Zhang et al., the expression of a novel miR153, was found to be influenced by both temperature and photoperiod in cucumber shoot apices. This novel miRNA was shown to target ERFs (ethylene responsive factors), which are key components of ethylene signaling pathways. Based on these findings, it is hypothesized that the novel miR153 may be involved in the intricate process of flower sex determination, potentially modulating the expression and activity of ERFs to regulate floral development and reproductive processes in cucumbers [183].

However, there is a limited understanding of the specific miRNAs involved in the regulation of flowering and fruit ripening in cucumbers, particularly in response to climatic factors. This knowledge gap represents a significant research opportunity to identify key miRNAs that are influenced by different climatic stresses and their effects on these developmental processes.

## 4. Future Prospects

**Figure 4:**
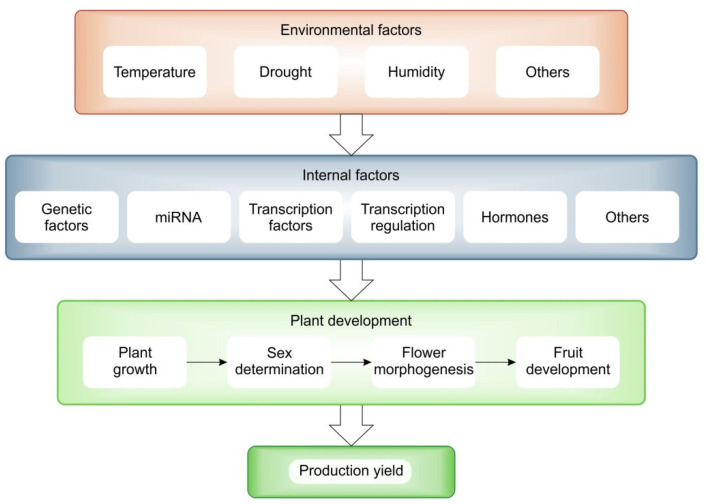
General diagram of the influence of climatic factors (red box) through internal regulatory factors (blue box) on plant growth and yield (green boxes).

Climate change is a natural process that cannot be stopped, but due to human activities the changes are faster and more severe. The observed environmental changes, mainly related to temperature increase and water scarcity, are particularly important in the case of plant crop production (Figure 4). Therefore, there is a need to focus on linking gene function to important agronomic traits. The development of high-throughput genetic techniques to study genomes and transcriptomes, together with the modern breeding technologies, provides potential for functional studies in cucumbers. The analysis of individual genes and their molecular functions is important, although the basic research is very laborious. In particular, the study of these genes in relation to important aspects that affect growth, development and yield in the context of how they are expressed under stress are of great importance. Knowledge of the function of genes can make a significant contribution to the development of modern varieties that are not only resistant to disease, but also able to withstand and adapt to the climatic conditions brought about by climate change. Plants can respond differently depending on where they are grown, the climate zone and the soil conditions. The ability to cope with adverse conditions in agriculture or horticulture is a power that will benefit humanity. Using techniques such as sequencing to learn about the regulation of the transcriptome and genome of crops will make it possible to learn not only about individual genes, but entire sets of the genes, how they are regulated and how they respond to changing climate conditions. Modern analytical technologies and the knowledge gained from gene regulation will make it possible to breed cucumbers well to produce sufficient yields to feed the world’s growing population.

## Data Availability

Not applicable.
